# Quantifying and Numerically Representing Recharge and Flow Components in a Karstified Carbonate Aquifer

**DOI:** 10.1029/2020WR027717

**Published:** 2020-11-04

**Authors:** P. Schuler, L. Duran, P. Johnston, L. Gill

**Affiliations:** ^1^ Department of Civil, Structural and Environmental Engineering Trinity College Dublin Dublin Ireland; ^2^ Irish Centre for Research in Applied Geosciences (ICRAG) Dublin Ireland

**Keywords:** karst, groundwater, time series analysis, signal decomposition method, pipe network model, Ireland

## Abstract

Karstified carbonate aquifers are highly heterogeneous systems characterized by multiple recharge, flow, and discharge components. The quantification of the relative contribution of these components, as well as their numerical representation, remains a challenge. This paper identifies three recharge components in the time and frequency domain. While the analysis in the time domain follows traditional approaches, the analysis of the power spectrum allows frequencies associated with specific spectral coefficients and noise types to be distinguished more objectively. The analysis follows the presented hypothesis that the different frequency‐noise components are the result of aquifer heterogeneity transforming the random rainfall input into a sequence of non‐Gaussian signals. The distinct signals are then numerically represented in the context of a semidistributed pipe network model in order to simulate recharge, flow, and discharge of an Irish karst catchment more realistically. By linking the power spectra of the modeled recharge components with the spectra of the spring discharge, the information usually gained by classical performance indicators is significantly widened. The modeled spring discharge is well matched in the time and frequency domain, yet the different recharge dynamics explain the signal of the aquifer outlet in different noise domains across the spectrum. This study demonstrates the conjunctive use of frequency analysis in conceptualization of a hydrological system together with modeling and evaluation.

## Key Points

- Hydrograph analysis was coupled with frequency and noise analysis to identify recharge dynamics in a karst spring discharge time series
- Recharge, flow, and discharge were modeled in a semidistributed pipe network model considering aquifer heterogeneities and noise domains
- A new model evaluation approach using noise analysis provided information on the individual contribution of the internal flows

## 1 Introduction

Karstified carbonate aquifers are highly heterogeneous geological formations characterized by multiscale temporal and spatial hydrological dynamics related to a primary (matrix), secondary (tectonic), and tertiary (dissolution) porosity (White & White, 2005) with associated permeabilities. The rock openings may constitute a continuum ranging between voids (>10 μm) up to accessible conduits or caves. Yet permeabilities are generally categorized in terms of two or three flow and recharge components (Atkinson, 1977; Kiraly et al., 1995; White & White, 2005). Recharge may concentrate quickly and directly via highly localized features into the conduit domain or diffusely, occurring over a larger area entering the low permeability fissured matrix blocks rather slowly (Geyer et al., 2008). Depending on the evolutional state of a karst aquifer (Kaufmann, 2002; Kovács et al., 2005), diffusely recharged groundwater may travel through different sized openings, from small fissures toward conduits, being subject to different flow dynamics en route ranging between laminar and fully turbulent flow (Giese et al., 2018).

No method exists to measure groundwater recharge directly. Indirect methods for quantifying recharge rely on chemical measurements (e.g., tracers) and/or physical measurements (e.g., water content and water table fluctuation) (Ireson & Butler, 2013), which have limited applicability in the context of heterogeneous karstified limestone. However, the dynamics of groundwater recharge and flow impact on the time‐amplitude signal of a hydrograph, most noticeably on its stable part of the recession. On a single rainfall event, the entire recession can be subdivided into individual recession components, such as a flood recession (quick concentrated component) and baseflow recession (slow diffuse component) (Geyer et al., 2008; Kovács et al., 2005). Hence, such event‐based recession analysis allows conclusions to be drawn with respect of the recharge dynamics (Mangin, 1975).

To widen the perspective of event‐based approaches, the analysis of a continuous time series of hydrographs may infer qualitative or semiquantitative information on global recharge and flow components. In general, hydrological time series analysis (and associated modeling) may be considered as the study of stochastic processes where a given signal contains, among others, an independent random component and the stochastic dependence (Yevjevich, 1993). Autocorrelation of time‐amplitude or time‐frequency signals may identify distinct memory effects related to different flow components (fast through to slow) (Jemcov & Petrič, 2010; Mangin, 1984; Padilla & Pulido‐Bosch, 1995). Further, based on the premise that signals within a time series can be differentiated by different frequencies (Holko et al., 2013), frequency analysis has shown that individual frequency components of a power spectrum of spring hydrographs and/or chemographs may provide useful information concerning the intrinsic structure of karst aquifers (Duran, 2015; Duran et al., 2020; Fournillon, 2012; Massei et al., 2007; Mathevet et al., 2004).

The Fourier spectrum of a stochastic signal/process represents the distribution of energy/variance *E* of the process depending on the frequency *ω*, where by *E* = *ω*^*β*^. The spectral coefficient, *β*, (defined by the frequency range slopes in log‐log space) is related to the Hurst exponent *H* that describes the persistence of a statistical phenomenon (Schroeder, 1991) which is related to the long‐term memory of natural systems and their observed time series (Hurst, 1951; Mandelbrot & Wallis, 1968). Hence, this paper proposes a method to detect and quantify different recharge and flow dynamics by (1) Gaussian and non‐Gaussian noise‐frequency components in the power spectrum of a karst spring, (2) assessing the information contained in the identified noise and Fourier spectra, and (3) representing these recharge and flow dynamics in a numerical model in order to compare the simulated dynamics. This paper hypothesizes that the deviations in noise across the spectra between rainfall and spring discharge are the result of aquifer heterogeneities, which can be numerically represented reasonably well by the proposed modeling approach.

Given the relevance (Hartmann et al., 2014) but at the same time complexity of (semi) distributed karst groundwater flow modeling (Sauter et al., 2006; Teutsch & Sauter, 1991), related modeling approaches are continuously evolving. This is crucial given the different flow dynamics in karst aquifers as well as the associated heterogeneous transport mechanisms of contaminants (Ghasemizadeh et al., 2012). However, distributed karst groundwater flow models do not usually tend to directly incorporate the real heterogeneity of recharge dynamics in terms of previously quantified components. The importance of representing the heterogeneity of recharge is addressed in this paper improving the pipe network modeling approach. Pipe network models have previously shown to excel well in terms of head dynamics (Gill et al., 2013; Kaufmann et al., 2016; McCormack et al., 2017; Vuilleumier et al., 2019) as well as overall discharge dynamics (Chen & Goldscheider, 2014; Schuler et al., 2018). Therefore, this study attempts to represent the different recharge and flow components that have been identified using noise analysis in a pipe network model for an autogenic spring catchment in Ireland using the software InfoWorks ICM (Version 7.0.5., Innovyze Ltd., Wallingford, UK). The performance of the model is then evaluated applying classical performance indicators, as well as an innovative approach of comparing the noise types between the modeled and observed spring discharge and the established recharge input time series. In this way, additional information is gained, which is considered useful with respect to improvements to this modeling approach.

## 2 Materials and Methods

### 2.1 Study Area

**Figure 1:**
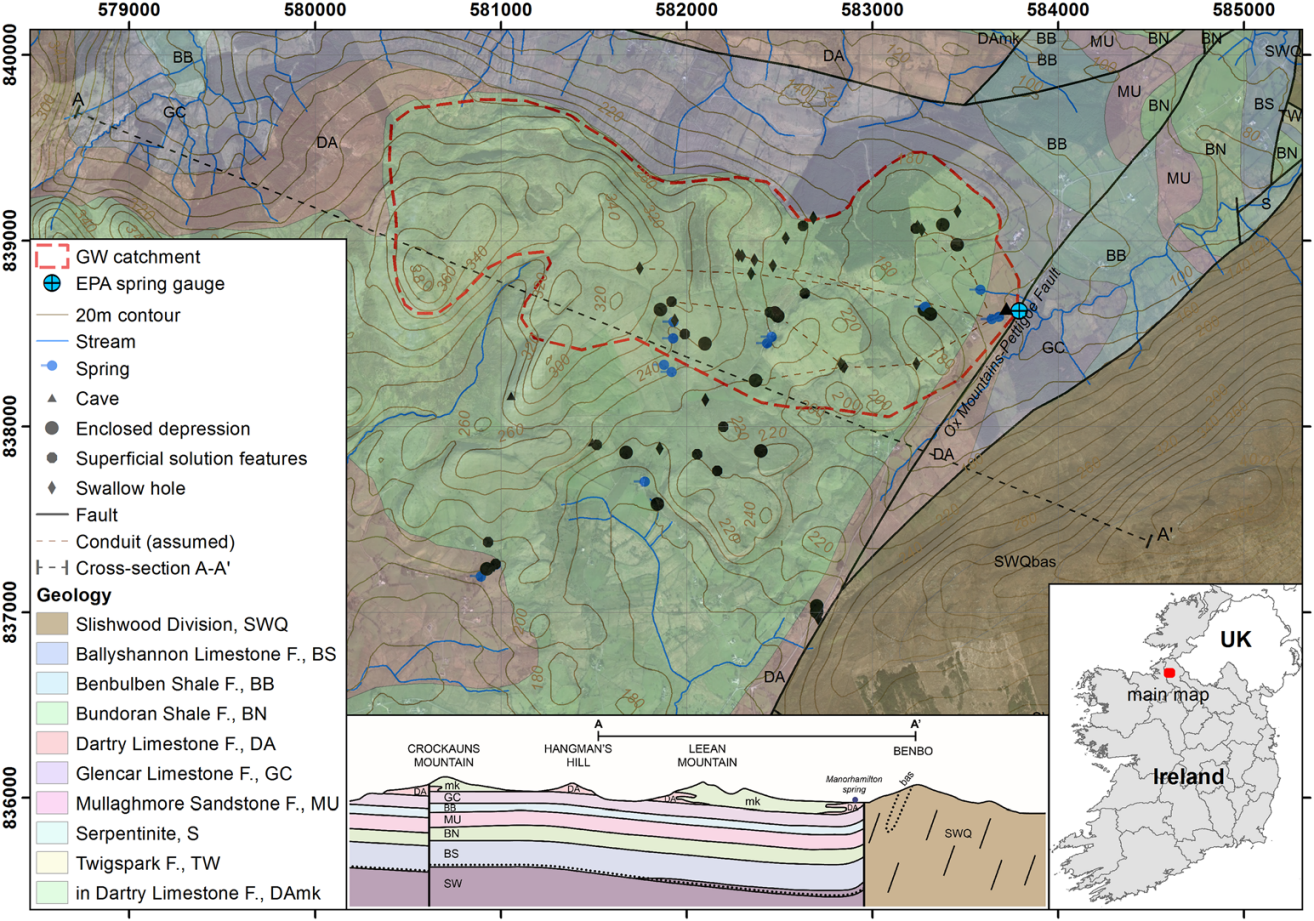
Geological map of the groundwater catchment of Manorhamilton, including karst features and the Cross Section A‐A′. Cross section modified and geology after GSI (2018) and MacDermot (1996).

The Manorhamilton spring catchment ranges between 112 m above sea level (masl) at the spring and 392 masl at Mt. Leean in the west, covering 3.6 km^2^ (Figure 1). The topography of the catchment is shaped by rounded or hummocky hills with grassland, covering largely chert‐free forms of limestones occurring as mudbanks (MacDermot, 1996). In the upper part, peat covers the outcrop. The catchment is highly karstified including many swallow holes and minor springs that act as epikarst discharge. During the hydrological years 2010 to 2018 the average annual rainfall was 1,567 mm (1,023 mm effective rainfall) and the average annual air temperature was 9.1°C.

The entire catchment is underlain by Lower Carboniferous limestones originating from a period of shelf sedimentation with local development of mudbank limestones of Viséan age, namely, the Dartry formation (DA), and specifically Mudbank Limestones (mk) within the Dartry Limestone formation (DAmk). The dominant facies of the Dartry Limestone formation is massive to thick‐bedded, mostly very fine grained and dark wackestone, with bedding by bands of nodules of irregular chert. Mudbanks occur within the Dartry Formation at its base in contact with the Glencar Limestone formation which is where the study area is located. The regional geology is very variable including many faults. The catchment is bound to the east by the Ox Mountains‐Pettigoe Fault (OMPF) and fine‐grained granoblastic psammitic paragneiss (Slishwood Division, Psammitic Paragneiss [SWQ]) of Proterozoic age (Figure 1) (MacDermot, 1996) controlling the occurrence of the contact spring Manorhamilton. The Dartry limestone formation (DAmk) forms the aquifer discharging at Manorhamilton spring, above the low‐permeable Glencar Limestone formation (GC), which both dip gently to the south/southeast (Figure 1). Groundwater flow follows roughly the topography and dip. Tracer tests confirmed a very shallow vadose zone and conduit transport velocities of 88 to 257 m/h. The aquifer is classified as a “conduit‐dominated regional important aquifer (Rkc)” (GSI, 2016). The mapped swallow holes indicate the presence of west‐east aligned conduits.

### 2.2 Data

The Irish Environmental Protection Agency (EPA) has been monitoring the discharge of Manorhamilton spring at 15 min intervals since April 2009, although the time series includes major data gaps. The data are freely accessible on the EPA HydroNet website (http://www.epa.ie/hydronet), and they were aggregated to an hourly time step using the software R (Version 3.6.2, cran.r‐project.org) and RStudio (Version 1.2.5019, www.rstudio.com).

A tipping bucket ARG100 rain gauge (Environmental Measurement Ltd., North Shields, UK) attached to a Rainlogger Model 3002 (Solinst Canada Ltd., Georgetown, Canada) data logger was installed at the spring to record rainfall between 15 December 2017 and 4 June 2019. Further climate data were obtained from the MetEireann station Markree, located at 34 masl, 19 km southwest of Manorhamilton spring observing daily rainfall (mm), and maximum and minimum daily air temperature (°C). Evapotranspiration (ET) was estimated following Hargreaves (1994) and the empirical parameters *H*_*A*_ (0.00197) and *H*_*E*_ (0.512), which were adjusted to the regional context by fitting the daily ET estimated for Markree against the estimated ET of a fully equipped weather station in western Ireland (Schuler et al., 2018) using the lowest root‐mean‐square error (RMSE) (Berti et al., 2014).

### 2.3 Methodology

#### 2.3.1 Characterization of Spring Discharges

For the characterization of the average aquifer dynamics, a representative average recession curve, that is, master recession curve (MRC) (Forkasiewicz & Paloc, 1967), was established using the software RC (Version 4.0, HydroOffice, Mojtin, Slovakia, https://hydrooffice.org) and Microsoft Excel (Redmond, WA, USA). The MRC was constructed by plotting rainfall and discharge and then summing up multiple recession segments of a hydrograph during which there was no visible rainfall (recharge) impact on the signal using MS Excel. The MRC was then related to rainfall, infiltration into and storage within the aquifer (El‐Hakim & Bakalowicz, 2007; Mangin, 1975). The method characterizes karst aquifers using two parameters, the infiltration delay *i* (describing the infiltration conditions, i.e., slow vs. fast) and the regulating power *k* (describing the mean hydraulic residence time of groundwater in the phreatic zone) which classifies karst springs into five groups. The approach is well suited for an overall characterization of a karst aquifer, although a limitation is the use of daily data, which may cause a loss of detail for fast responding systems, such as the Manorhamiltion spring. The method divides the recession into an upper homographic function *ψ*(*t*) in which recharge impacts on spring discharge, and a lower baseflow function *φ*(*t*) following Maillet (1905) representing drainage of the phreatic zone not being impacted by rainfall. Fitting of, first *ψ*(*t*) and then *φ*(*t*) was carried out visually and then numerically by matching the sum of *ψ*(*t*) and *φ*(*t*) against the MRC using the Nash‐Sutcliffe efficiency (NSE) (+0.990). The baseflow can be conceptualized to relate to diffuse groundwater recharge and flow (Geyer et al., 2008), and its recession constant *k*′ (h^−1^), *k* (day^−1^), respectively, following Maillet (1905). Using this *k* value, a continuous baseflow signal *Q*_*bf*_ was established using a digital recursive filter (Eckhardt, 2005; Rimmer & Hartmann, 2014) with the daily recession constant *a* = exp(−*k*) and the hourly recession constant *a*′ = *a*^1/24^. The maximum of the baseflow index (*BFI*_*max*_), that is, the ratio of baseflow to observed discharge/flow, was optimized by fitting *Q*_*bf*_ against the exponentially separated low‐flow components of the discharge time series and minimizing the RMSE.

#### 2.3.2 ACF and CCF Functions

Autocorrelation and cross correlation are based on the assumption that time series reflect bivariate stochastic processes that are stationary (Box & Jenkins, 1976).

A finite time series with *N* observations has the autocorrelation *r*_*k*_ at the lag *k*:

(1) $$r_{k}=\frac{c_{k}}{c_{0}}$$

with the autocovariance function,

(2) $$c_{k}=\frac{1}{N}\sum_{t=1}^{N-k}\left(z_{t}-\bar{z}\right)+\left(z_{t+k}-\bar{z}\right),k=0,1,2,\ldots$$

and the variance function,

(3) $$c_{0}=\frac{\sum_{t=1}^{N}{\left(z_{t}-\bar{z}\right)}^{2}}{N}$$

with the mean $\bar{z}$ of the time series.

Autocorrelation describes the time series' persistence, which is the tendency for the magnitude of an event to be dependent on the magnitude of the previous event. Accordingly, persistence is present if data in the time series are dependent on each other, describing a memory effect of a system (Machiwal & Jha, 2006). Memory effects are interpreted as different storage systems releasing different flow components (fast and slow) within the aquifer (Jemcov & Petrič, 2010; Mangin, 1984; Padilla & Pulido‐Bosch, 1995; Panagopoulos & Lambrakis, 2006). Further, the shape of the Autocorrelation fuction (ACF) provides insight into the randomness of a signal. Since the introduction of autocorrelation in karst hydrology, a value *r*_*k*_ = 0.2 has been widely used to distinguish a memory effect from white noise (Mangin, 1984), while other authors applied the significance level of 0.05 (Jemcov & Petrič, 2010). This is relevant for the assessing how the karst aquifer transforms the rainfall signal toward an (at least) partly structured signal observed at the spring outlet.

Cross correlation quantifies the strength of the linear relationship between two time series (input‐output) of a karst aquifer (Angelini, 1997; Labat et al., 2000; Larocque et al., 1998; Massei et al., 2006; Mathevet et al., 2004; Padilla & Pulido‐Bosch, 1995). For a discrete number of *n* pairs of values (*x*_1_, *y*_1_), (*x*_2_, *y*_2_), … (*x*_*n*_, *y*_*n*_) the cross covariance *c*_*xy*_(*k*) at lag *k* is estimated by

(4) $$c_{\mathrm{xy}}\left(k\right)=\left\{\begin{array}{c} \frac{1}{n}\sum_{t=1}^{n-k}\left(x_{t}-\bar{x}\right)\left(y_{t+k}-\bar{y}\right),k=0,1,2,\ldots \\ \frac{1}{n}\sum_{t=1}^{n+k}\left(y_{t}-\bar{y}\right)\left(x_{t-k}-\bar{x}\right),k=0,-1,-2,\ldots \end{array}\right)\;$$

where $\bar{x}\;\;\text{and}\;\bar{y}$ are the means of series *x* and *y*, respectively. The cross correlation *r*_*xy*_(*k*) at lag *k* is estimated by

(5) $$r_{\mathrm{xy}}\left(k\right)=\frac{c_{\mathrm{xy}}\left(k\right)}{s_{x}s_{y}},k=0,\pm 1,\pm 2,\pm \ldots$$

with the standard deviation *s*_*x*_ and *s*_*y*_ of series *x* and *y*, respectively.

The ACF and cross‐correlation function (CCF) was executed using the package “stats” (Version 3.6.2) for the software R (Version 3.6.3, www.r‐project.org) (R‐Core‐Team, 2019).

#### 2.3.3 Frequency and Noise Analysis

Frequency analysis evaluates a time‐amplitude signal in terms of its spectrum of frequencies or related quantities such as energies. In the context of karst hydrograph analysis, the different recharge and flow components of a karst aquifer translate into different frequency components at the spring outlet and so should be contained within the final spectral signature of the spring discharge. Within a log‐log spectral density plot of spring discharge, the slope of each frequency segment bounded by break points can be approximated by a linear regression to yield the spectral coefficient *β*, which relates to a noise domain (Beier & Hardy, 1996, in Fournillon, 2012; Mathevet et al., 2004; Schroeder, 1991) and where

−1 < *β* < 1 relates to Gaussian noise: Data pairs are stochastic and independent from each other, −*β* = 2*H* − 1;
- *β* = 0 relates to white noise: That is, the power spectrum is independent of frequency, and so no information is contained within the spectrum, *H* =  − 0.5;
- *β* = −1 relates to pink noise or 1/*f* noise: describing ubiquitous natural phenomena on a large scale in time and space, present in many time series;
−3 < *β* < −1 relates to Brownian noise, −*β* =  − 2*H* + 1:
  −2 < *β* < −1 corresponds to antipersistent Brownian noise with poor correlation between data pairs;
  - *β* = −2 corresponds to red noise (or brown noise following Schroeder, 1991) where the data pairs are independent from each other but follow a statistical law, Brownian motion;
  −3 < *β* < −2 corresponds to persistent Brownian noise with a memory effect and high correlation between data pairs;
- *β* < −3 relates to black noise: widespread occurring signal that is not stochastic but structured, and the signal may represent exceptional events and catastrophes like floods and droughts.

The change of slopes is assumed to coincide with a change in the discharge dynamics (e.g., low, intermediate, and high) (Duran et al., 2020), whereas the numeric value of *β* may be linked to different karst aquifer dynamics.

Significant changes in the power spectrum may be identified following Pettitt (1979), which is rank‐based nonparametric statistical test method frequently applied in hydrology (Mallakpour & Villarini, 2016; Rougé et al., 2013; Rybski & Neumann, 2011) and karst hydrogeology (Dufoyer et al., 2018). The Pettitt test compares two samples and therefore creates two segments of the original time series. Hence, in order to identify multiple break points, the method was applied iteratively on the spectrum until no statistically significant breakpoint with a *p* value <0.05 was generated in R (Pohlert, 2016).

Each resulting frequency segment was then characterized by *β* and the type of noise, which allowed the entire power spectrum to be subdivided into subsequent regions of common noise. In this way, the power spectrum was objectively separated into different slopes, based on noise. This paper argues that the different types of noise and associated frequency segments are the result of flow and dynamics, accounting potentially for a slow, intermediate, and fast aquifer response.

The power spectrum (squared magnitude of the Fourier transform) of observed time series was computed based on the fast Fourier transform (FFT) implemented in R based on Cooley and Tukey (1965) (see the supporting information for the code http://doi.org/10.5281/zenodo.3961605).

#### 2.3.4 Pipe Network Model

Pipe network models have proved to be a most appropriate modeling tool in the context of low lying catchments with a shallow vadose zone (Gill et al., 2013; McCormack, 2014; Morrissey et al., 2020; Schuler et al., 2018) and have also been used in high elevated alpine systems (Chen & Goldscheider, 2014; Kaufmann et al., 2016; Mayaud et al., 2019; Vuilleumier et al., 2019).

In this study, semidistributed groundwater flow is modeled using the urban‐drainage software InfoWorks ICM (Version 7.0.5., Innovyze Ltd., Wallingford, UK), similarly to the above cited related references. However, the novel approach required innovation in order to match the functioning of this software with the conceptual understanding of a karst aquifer. The modeling principle comprises two fundamental aspects: (1) generating different recharge components originating in subcatchments, which then infiltrate into (2) a semidistributed network of pipes in which flow is solved following different equations. Flow is modeled in 1‐D either in “permeable pipes” following Darcy's law or in “full pipes” modeling shallow water flow and pressurized pipe flow following the Saint‐Venant equations:

(6) $$\frac{\mathrm{\delta A}}{\mathrm{\delta t}}+\frac{\mathrm{\delta Q}}{\mathrm{\delta x}}=0$$

(7) $$\frac{\mathrm{\delta Q}}{\mathrm{\delta t}}+\frac{\delta }{\mathrm{\delta x}}\left(\frac{Q^{2}}{A}\right)+\mathrm{gA}\left(\cos\theta \frac{\mathrm{\delta y}}{\mathrm{\delta x}}-S_{0}+\frac{Q\left|Q\right|}{K^{2}}\right)=0$$

with the discharge *Q* (m^3^/s), the cross‐sectional area *A* (m^2^), the acceleration due to gravity *g* (m/s^2^), the angle of bed to horizontal (°) *θ*, the bed slope *S*_0_, and the conveyance *K*. The conveyance is expressed by the Colebrook‐White expression (Kellagher, 1981). The transition between shallow water flow and pressurized pipe flow is enabled by using a “Preissmann slot” (e.g., Zhang & Lerner, 2000), which is a conceptual vertical and narrow slot that provides a conceptual free surface condition when the water level exceeds the top of the conduit. In comparison, other equations commonly used to solve turbulent groundwater flow in conduits follow Darcy‐Weisbach (Ford & Williams, 2007), Colebrook‐White (Giese et al., 2018) or Manning‐Strickler (Binet et al., 2017).

**Figure 2:**
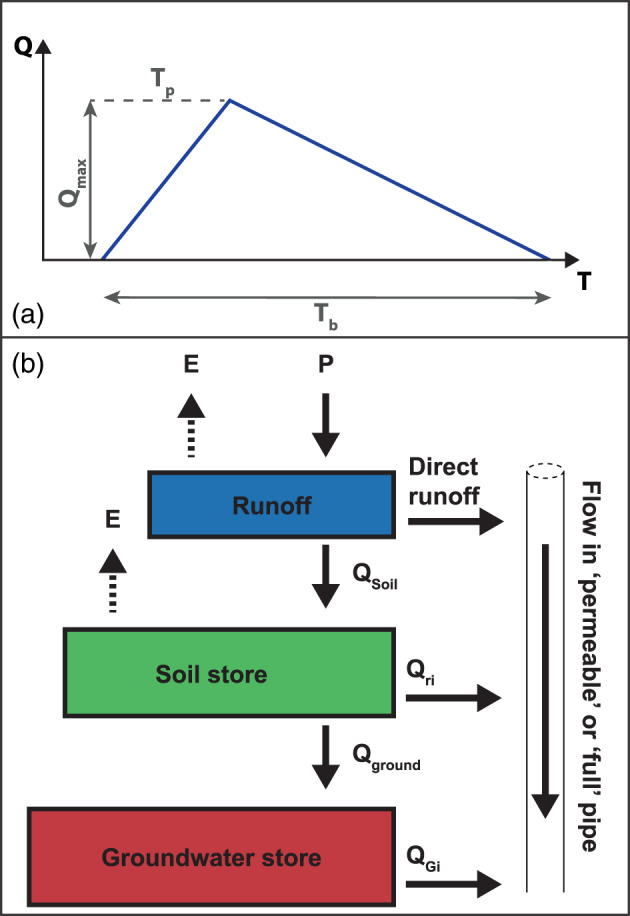
(a) Unit Hydrograph Model consisting of the time to peak flow *T*_*p*_, the total runoff time *T*_*b*_, and the discharge *Q* in time *T* for the runoff component and (b) schematic of flows (runoff, soil store inflow, and groundwater store inflow) generated in InfoWorks ICM and entering the pipe network, before finally discharging at the outlet (spring).

Recharge entering the pipe network is fast/concentrated, intermediate, or slow/diffuse. The fast component is expressed through a triangular runoff model (Figure 2a) coupled with the curvilinear Soil Conservation Service (SCS) hydrograph (McCuen et al., 2002).

The intermediate (*Q*_*ri*_, Figure 2b) and slow (*Q*_*Gi*_, Figure 2b) network recharge component are modeled using a combination of two quasi‐linear reservoirs: rainfall (*P*) that is not subject to evaporation (*E*) and runoff enters the soil store (*Q*_*Soil*_). From there, the different fractions are subject to *E*, and then discharge into the conduit network (*Q*_*ri*_) while the remainder enter the lower groundwater store (*Q*_*Ground*_). The groundwater store discharges into the conduit network (*Q*_*Gi*_). Hence, any of the three recharge components can directly enter the pipe network (in which flow is then computed following the above mentioned equations) before discharging at the spring.

The calibration process includes the following steps carried out in the following order: (1) matching the overall water balance by evaluating the volume conservation criteria (VCC); (2) matching the timing and slope of recessions of the fast, intermediate, and slow recharge component against the recession constants *k'* obtained from the MRC; and (3) matching the simulated discharge against the observed discharge (performance) using first, the Kling‐Gupta efficiency (KGE) (Gupta et al., 2009; Kling et al., 2012) and second, the NSE. Finally, the power spectrum of the observed and simulated time series, including their spectral coefficients, provided the basis for a more thorough discussion. The warm‐up, calibration, and validation periods are 15 to 31 December 2017, 1 January to 31 December 2018, and 1 January to 4 June 2019, respectively.

## 3 Results

### 3.1 Aquifer Characterization

**Figure 3:**
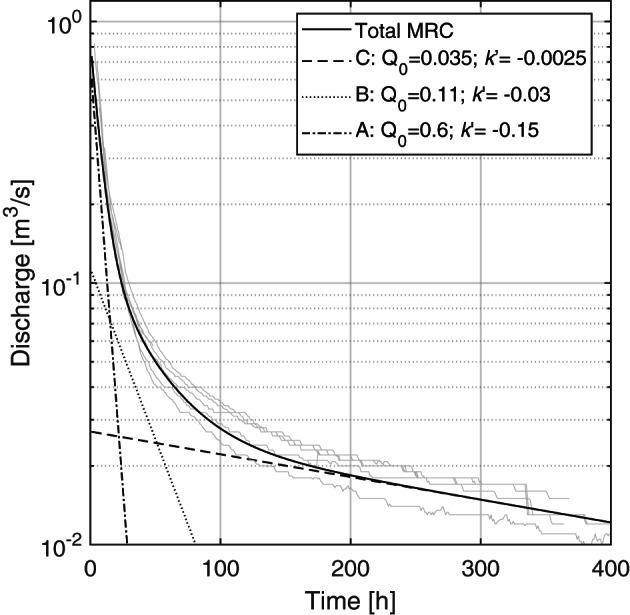
Master recession curve of Manorhamilton spring (h) split into three contributing linear reservoirs. The observed recessions used to construct the MRC are displayed in gray.

The spring discharge between April 2009 and June 2019 ranges between 0.004 and 2.08 m^3^/s with a mean of 0.14 m^3^/s. Figure 3 shows the MRC of hourly discharge determined for the Manorhamilton spring, decomposed into three linear reservoirs on a semilogarithmic plot following Maillet (1905) where “A” is the major fast recession (*k*′ = − 0.15 h^−1^), “B” is the intermediate recession (*k*′ = − 0.03 h^−1^), and “C” is the minor slow recession (*k*′ = − 0.0025 h^−1^). The range of *k*′ values represents the dynamic recharge and discharge of the system. The maximum “low‐flow” contribution (“C”) is 0.035 m^3^/s. Following El‐Hakim and Bakalowicz (2007) and Mangin (1975), the aquifer exhibits a relatively low regulating power (i.e., the rainfall input is hardly transformed en route to the spring outlet), the mean hydraulic residence time is 0.26 years or 95 days, and it is classified as aquifer “with a well karstified infiltration zone and an extended conduit network ending into a flooded phreatic zone”.

### 3.2 ACF and CCF

**Figure 4:**
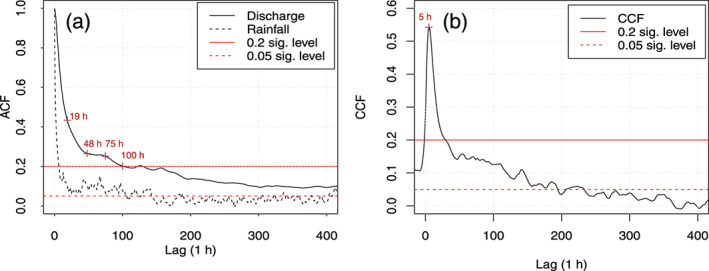
(a) Autocorrelation function (ACF) of hourly rainfall and spring discharge and (b) cross‐correlation function (CCF) between hourly rainfall and spring discharge and the significance level of 0.2 and 0.05 respectively. The red labels in the plots highlight changing slopes (ACF) or the peak (CCF).

The ACF of the rainfall time series shows a very quick loss in memory to below the 0.2 and toward the 0.05 significance level (Figure 4a) indicating randomness.

The ACF of spring discharge suggests a rapid loss of memory reaching <0.2 after 100 h (Figure 4a). The ACF suggests different changing slopes, namely, at approximately 19, 48 and 75 h, which can be interpreted as evidence of different flow contributions. The CCF between hourly rainfall and spring discharge peaks at 0.54 and a lag of 5 h, which suggests a very rapid response of discharge to rainfall, accompanied by a rapid decline of the CCF.

The results of the ACF and CCF therefore indicate a very responsive spring discharge signal and a low storage aquifer. The aquifer seems to exhibit a relatively low filtering effect of the random rainfall input.

### 3.3 Frequency and Noise Analysis

**Figure 5:**
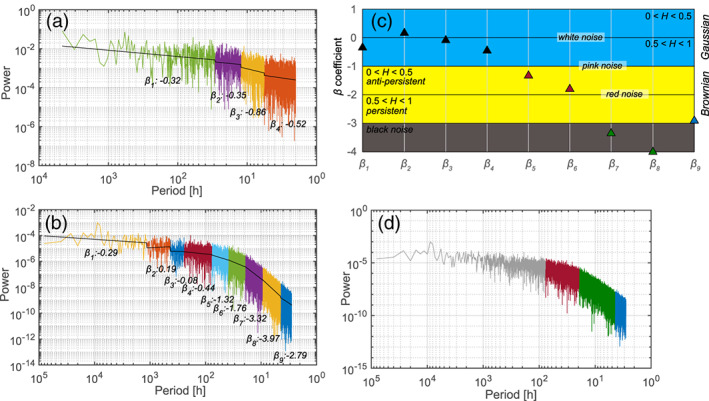
Power spectrum (h) of (a) hourly rainfall and (b) spring discharge, and frequency segments following the Pettitt method with associated spectral exponents *β*, (c) noise types of the spectral coefficients *β*_1_ to *β*_9_ related to frequency segments of the spring discharge power spectrum, including the Hurst exponent *H*, and (d) aggregated low (red), intermediate (green) and high (blue) frequency components of spring discharge. The gray frequencies correspond to Gaussian information.

The power spectrum of the entire hourly rainfall yields three significant breakpoints and four spectral coefficients *β* (Figure 5a). All spectral coefficients are > −1, hence associated with the Gaussian domain. The deviation from random noise (*β* = 0) suggests the presence of statistical dependence, presumably related to seasonal climatic effects with a cyclicity of a few days or weeks.

The power spectrum of the spring discharge signal (2009 to 2018) yields eight significant breakpoints with spectral coefficients *β* ranging between 0.19 and −3.97 (Figure 5b). The slope of the resulting frequency segments yields *β* coefficients which relate to different noise types (Figure 5c). Within the low frequency end, the spectral coefficients *β*_1_ to *β*_4_ (≤0.0130 Hz; ≥77.2 h) are ≥ −1 and therefore relate to Gaussian noise. The coefficients *β*_5_ and *β*_6_ (0.0130 to 0.0517 Hz; 77.2 to 19.4 h) relate to antipersistent Brownian noise, while *β*_9_ (0.2246 to 0.500 Hz; 4.5 to 2 h) relates to persistent Brownian noise. The coefficients *β*_7_ and *β*_8_ (0.0517 to 0.2246 Hz; 19.4 to 4.5 h) correspond to black noise. A large difference between *β*_6_ and *β*_7_ of −1.56 and between *β*_8_ and *β*_9_ of −1.18 separates the spectral coefficients between Brownian noise and black noise.

While the rainfall power spectrum completely corresponds to Gaussian noise, the spring discharge power spectrum only relates partly to Gaussian noise. Therefore, the non‐Gaussian noise of the spring discharge power spectrum characterizes more clearly the filtering effect of the aquifer (transformation of the Gaussian rainfall input), as this noise deviates from the Gaussian rainfall input. The non‐Gaussian range of the spring discharge power spectrum comprises three frequency components, namely, a low‐frequency component of antipersistent Brownian noise (*β*_5_ to *β*_6_), an intermediate‐frequency component of black noise (*β*_7_ to *β*_8_), and a high‐frequency component of persistent Brownian noise (*β*_9_) (Figure 5d).

Any periods >77.2 h (Gaussian) are considered as long‐term or slow response of the system, which cannot be distinguished from the Gaussian rainfall input. Instead, only frequencies between 2 and 77.2 h are considered to contain more structured information which is hypothesized to result from the aquifer transforming the rainfall signal. This range of structured information relates to relatively high frequencies which seems plausible in the context of the recharge processes for this rapidly responding system.

### 3.4 Pipe Network Model

The aim of the pipe network model was to simulate the overall spring discharge by accounting for the different recharge and flow components previously identified—that is, to integrate the heterogeneous recharge dynamics prior to simulating groundwater flow.

**Figure 6:**
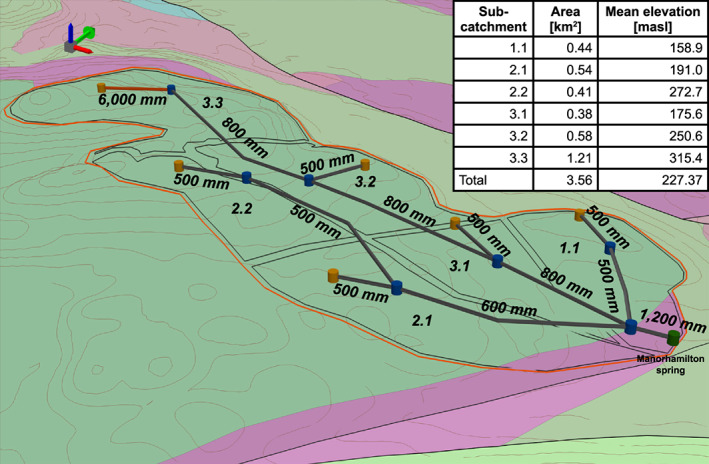
Pipe network model consisting of six subcatchments (1.1 to 3.3) contributing recharge components via “manholes” (yellow cylinders) into a permeable (red) or full (dark gray) pipes connected by manholes (blue cylinders) finally discharging at Manorhamilton spring (green cylinder). The background shows the geology, structure and topography as illustrated in Figure 1.

The model domain was set up to comprise of six subcatchments (Figure 6) aligning along the assumed conduits, each resembling an area of relatively uniform land cover. Subcatchments 1.1 to 3.2 drain into a full pipe, while Subcatchment 3.3 drains into a permeable pipe reflecting the dampened recharge affected by peat cover (Figure 6a). The final discharge at the spring is the sum of the fast and concentrated recharge (runoff), the intermediate recharge (*Q*_*ri*_), and the slow diffuse recharge (*Q*_*Gi*_,) (Figure 6b) which all individually enter the pipes with subsequent accumulated flow through the network (see section 2.3.4).

**Figure 7:**
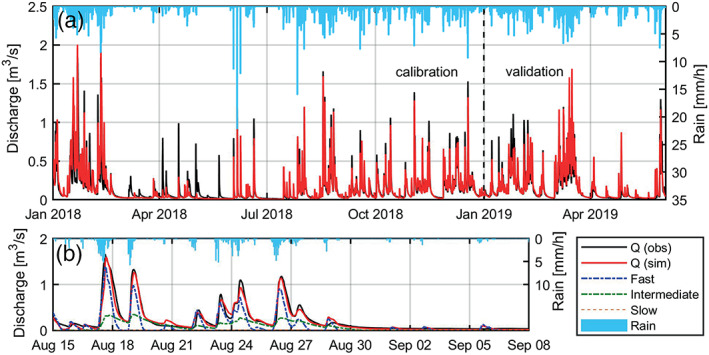
(a) Time domain results: Observed and modeled discharge of Manorhamilton spring and observed hourly rainfall during calibration and validation (*note: low modeling performance between March and June 2018 occurred due to underestimated rainfall related to faulty rain sampling in this period*) and (b) observed and modeled spring discharge between 15 August and 8 September 2018 as a result of the pipe flow dynamics as well as the three recharge components, namely, fast (“ICM runoff ”), intermediate (“ICM soil store”) and slow (“ICM Groundwater store”).

Within the time domain, the total simulated spring discharge matches the observed discharge well (Figure 7a). During calibration and validation, the NSE and KGE are 0.807 and 0.904, and 0.882 and 0.892, respectively (Table 1). The volume of the simulated discharge matches very well the volume of the observed discharge during calibration (+0.21%), while during validation, the simulated discharge exceeds the observed discharge by 9.04% which was assumed to be related to a period of inconsistent rainfall measurement during calibration.

**Table 1:** Performance of the Simulated Discharge Compared to the Observed Spring Discharge of Manorhamilton Spring (MCM = million m^3^)

Performance indicator	Calibration		Validation	
NSE	+0.807		+0.882	
KGE	+0.904		+0.892	
VCC	+0.020 MCM	+0.21%	+0.196 MCM	+9.04%

Figure 7b exemplifies the contribution and recessions of the three recharge components with regard to the overall observed and simulated discharge.

**Figure 8:**
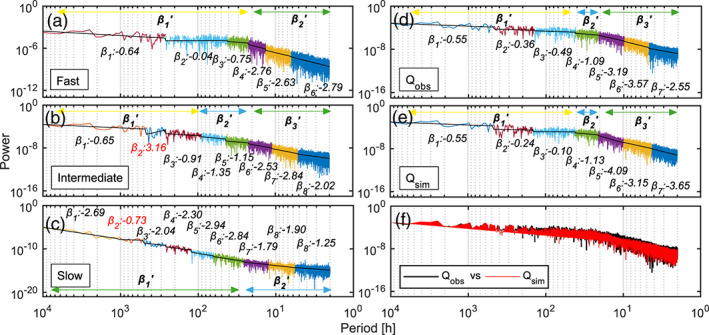
Frequency domain results: power spectrum of the simulated (a) fast, (b) intermediate, and (c) slow recharge component and power spectrum of the (d) observed and (e) simulated spring discharge, (f) overlapping power spectrum of the observed and simulated discharge, (a) to (e) contain the spectral coefficients *β* for each frequency range separated by break points. Further, coefficients *β* were aggregated to *β*_*n*_′ coefficients according to the common noise domain to yield information on the common underlying aquifer transformation processes (yellow = low frequency; blue = intermediate frequency; and green = high frequency).

The results in the frequency domain are discussed hereafter. Figures 8a–8e present the power spectrum of the three simulated recharge components and the observed and simulated spring discharge, including the spectral coefficients for each frequency segment separated by the Pettitt test. Periods of common noise types of the spectral coefficients were summarized into three groups to *β*_1_′, *β*_2_′, and potentially *β*_3_′ (low to high frequency) associated with either Gaussian (yellow), antipersistent (blue), or persistent/black (green) Brownian noise. Notably, *β*_2_ (red) in Figures 8b and 8c is considered as “artifacts” as they stand out considerably from their surrounding coefficients. The fast and intermediate components follow a typical concave power law decay, while the slow component follows a convex shape. The fast and slow components were aggregated to two *β*′ coefficients, covering less noise types than the intermediate power spectrum that consists of three *β*′ coefficients.

Figure 8f plots the power spectra of the observed and simulated discharge time series overlapped to increase the comparability. The simulated spectrum shows a very consistent course with regard to the observed spectrum, with a RMSE of 1.64 × 10^−7^.

**Figure 9:**
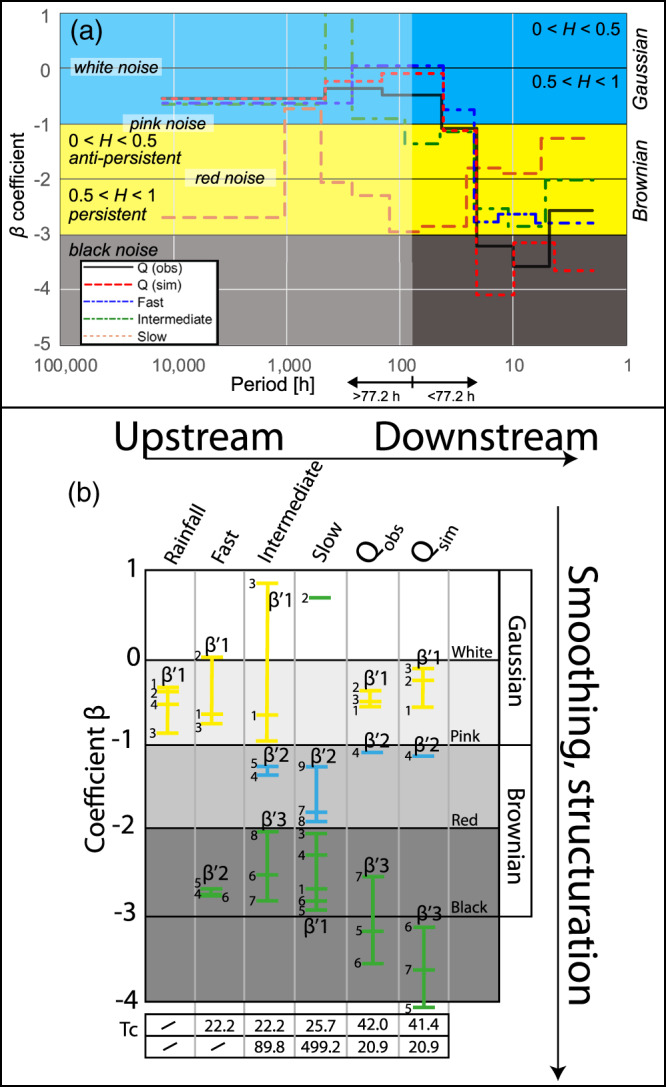
(a) Comparison of the spectral coefficients per associated period (h) between the observed and simulated discharge and the three recharge components (fast, intermediate, and slow), (b) structure matrix of all spectral coefficients occurring between 1 (low frequency) and 9 (high frequency) of observed and simulated time series across the noise domains. The individual spectral coefficients were aggregated to *β*_1_′ to *β*_3_′ associated wither with low (yellow), intermediate (blue), or high (green) frequencies. The cutoff (Tc) periods (h) are given for every signal analyzed on the bottom.

Figure 9a provides a more detailed analysis comparing the spectral coefficients of the three recharge input series, as well as the modeled and observed spring discharge signals across the same corresponding period (h). The subdivision of the power spectra of the modeled and observed spring discharge yielded seven *β* coefficients, which are summarized in Table 2, including their absolute and relative differences, on an illustrative basis for the more thorough analysis hereafter. Notably, the period investigated here is shorter than for the time series in Figure 5d, which explains a lower number of *β* coefficients, frequency components, and hence a less detailed result. Nevertheless, the trend of the *β* coefficients related to the observed spring discharge time series is very similar to the ones in Figure 5d.

**Table 2:** Spectral Coefficients and Their Absolute and Relative Difference of the Observed and Simulated Time Series

	*β*_1_	*β*_2_	*β*_3_	*β*_4_	*β*_5_	*β*_6_	*β*_7_
Observed	−0.55	−0.36	−0.49	−1.09	−3.19	−3.57	−2.55
Simulated	−0.55	−0.24	−0.10	−1.13	−4.09	−3.15	−3.65
Absolute difference	0.01	−0.12	−0.39	0.04	0.90	−0.43	1.10
Relative difference	0.00	0.50	3.90	−0.04	−0.22	0.13	−0.30

The comparison of the signals related to the simulated and observed spring discharge time series shows that the *β* Coefficients 1 to 3 (Gaussian noise) and 4 (antipersistent Brownian noise and low‐frequency component) are within the same noise domains and with similar coefficients. Above 140.2 h, the spectral coefficients (−0.55 and 0.36) are very well matched by the model (−0.55 and −0.24). The *β* Coefficients 5 and 6 related to both, the simulated and observed time series, correspond to black noise representing the intermediate frequency component. Yet the magnitude of the spectral coefficients differs by 0.90 and −0.43, respectively. However, a difference in noise types occurs in the high‐frequency range: While *β*_7_ of the simulated signal relates to black noise too, *β*_7_ of the observed signal relates to persistent Brownian noise. Hence, the results suggest that with regard to spring discharge, the model performs best in the low‐frequency range, deviating mainly in the very high frequency range between 2 and 4.4 h.

The analysis of the power spectra of the three modeled recharge signals yields additional results, focusing on the period to which non‐Gaussian noise applies (<77.2 h): In the very high frequency range (2 to 4.3 h), the fast and concentrated components have a *β* of −2.79, which matches very well to the corresponding *β* of −2.55 related to the observed discharge signal. Between 4.3 and 20.9 h, the *β* coefficients of the fast and intermediate recharge components deviate in similar magnitude from the corresponding *β* related to the observed spring signal. Between 20.9 and 42.0 h, the *β* related to the simulated discharge signal and the *β* of the intermediate recharge component perfectly match the *β* related to the observed signal. Above 42.0 h, the *β* coefficients of the intermediate and fast recharge components deviate again in a similar fashion from the *β* coefficients of the observed spring series. Notably, the *β*s of the slow recharge component exhibit the most pronounced deviations at any time across the entire spectrum.

Figure 9a suggests that at certain periods, the fast and intermediate recharge components match the observed spring signal very well in the frequency domain in terms of their spectral coefficients. At the same time, the slow recharge component deviates largely. This indicates that either the simulated slow recharge component does not capture well such the corresponding slow dynamics occurring in reality and/or there is an absence of any relevant or identifiable slow recharge dynamics in the natural system and, hence, no possibility to represent it numerically.

Figure 9b plots all spectral coefficients in the context of the hierarchical function of a karst aquifer (“upstream” to “downstream”) across the noise domains. All observed and simulated signals contain Gaussian antipersistent noise, except the slow recharge signal. The latter can be interpreted as the result of filtering of the slow recharge component, impacting mainly on low frequencies and, hence, removing Gaussian noise. The proportion of Gaussian noise in the simulated as well as observed discharge is likely to reflect the spectral signature of rainfall.

The intermediate frequencies (*β*_2_′, antipersistent between pink and red noise) of the simulated spring discharge appear to be a function of the soil store (intermediate recharge) and the groundwater store (slow recharge), matching the observed time series between 20.9 and 42.0 h. The high frequencies of the spring discharge are largely filtered: In fact, filtering is stronger than it appears for any of the recharge components (*β*_3_′ < −3), reflecting the conduit dominated nature of the karst aquifer. The high‐frequency noise types of the simulated spring discharge are controlled by the fast and intermediate recharge components, as indicated previously (Figure 5a).

The slow recharge component behaves differently, filtering the low frequencies to a larger extent than the high frequencies.

## 4 Discussion and Conclusion

The aim of this paper was to identify and quantify recharge components in karst spring discharge time series in order to then represent these components numerically in a pipe network model. The analysis of recharge components linked to groundwater flow within the power spectrum of the discharge signal is based on the principle that hydrological time series can be decomposed into an independent random component, the stochastic dependence, the periodicity, and the trend (Yevjevich, 1993). This paper evaluates the model across the full frequency domain, based on the aquifer heterogeneity transforming the random rainfall signal into relatable non‐Gaussian noise in the power spectrum of the spring. Hence, this paper suggests that the non‐Gaussian frequency range within a power spectrum can yield detectable information related to aquifer heterogeneities. The evaluation of the modeled dynamics and their power spectra and spectral coefficients indicates that some observed dynamics can be realistically represented in the model, while others cannot be represented.

The analysis of the time‐amplitude signal for the studied spring catchment in the west of Ireland shows that the aquifer storage is low and mean hydraulic residence times are <100 days related to the high degree of karstification, as indicated by the MRC analysis (El‐Hakim & Bakalowicz, 2007; Mangin, 1975). Corresponding to this, the memory loss of the spring discharge is rapid at 100 h at a significance level of 0.2, while changes in the slope were identified for approximately 48 and 75 h. Arguably, this significance level may be considered as arbitrary; however, the duration closely matches the results of the frequency and noise analysis: Any frequencies ≤0.0130 Hz or ≥77.2 h were interpreted as being the result of aquifer transformation of the rainfall input signal, and therefore, frequencies >0.0130 Hz or <77.2 h were considered to capture the aquifer impact's on the input signal.

A perfect separation of frequency components is challenged by the overlapping frequency domain of signals (Spongberg, 2000). At the same time, such objective separation is necessary in order to evaluate the distinct components. Within both, the time and frequency domain, three components were identified and conceptualized as the signature of recharge and flow processes. The fact that three exponential components could be fitted along the recession has been suggested theoretically to reflect symmetric rectangular blocks in a karst aquifer (Kovács & Perrochet, 2008). However, while fitting the exponential components is subjective to some extent, the approach presented here in the frequency domain increases objectivity by (a) establishing significant break points, (b) computing the spectral coefficient *β* for the frequencies bounded by these break points, (c) assigning noise types to the *β* coefficients, and finally, (d) grouping *β* coefficients of same noises to *β*′ coefficients representing a common dynamic in order to analyze them by type noise. In this way, two to three (non‐Gaussian) components were also identified. Notably, the intermediate recharge components contain more noise types than the fast and slow component, indicating that this simulated recharge dynamic covers a wider range of dynamics, which perfectly matches parts of the spectrum of the observed spring discharge. This conclusion suggests the hypothesis that the soil store (intermediate recharge) may mimic the functioning of an epikarst, which can act as a perched and shallow subsystem generating diffuse and concentrated flows (Aquilina et al., 2006; Perrin et al., 2003).

A limitation of this approach is the length of the time series required: The Pettitt test performs better in detecting change points with an increasing sample size (Xie et al., 2014), which is believed to be the reason why the number of change points detected decreases with sample size. Hence, it seems that the longer the time series, the higher will be the “resolution” in terms of change points, spectral coefficients, and (most importantly) potential information on recharge and flow components.

Overall, the simulated spring discharge matched very well the observed spring discharge, as indicated by the NSE and KGE. However, more information on the performance of the internal dynamics can be achieved. The frequency and noise analysis were applied on the observed as well as simulated time series to evaluate the recharge dynamics of the aquifer, and further, the pipe network modeling approach that incorporates the previously detected recharge and flow heterogeneity. The analysis focused on the non‐Gaussian noises. Overall, the power spectrum of the simulated time series matches the power spectrum of the observed time series well. In fact, the spectral coefficients are very well matched for certain periods by the fast and intermediate recharge components. In turn, the spectral coefficients of the slow (diffuse) recharge deviates across the entire spectrum, indicating that the representation of this component can be improved, for example, by modifying the discharge equation of the groundwater store. Hence, the methodology presented widens the perspective of the traditional performance criteria (e.g., KGE and NSE) to enable the interpretation of discrete recharge and flow components using noise analysis. In fact, the here presented approach is a new way to assess the model performance in a detailed, quantitative way, which suggests new perspectives of calibrating models in the future.

The lower performance of the slow recharge component may be explained by the functioning of the model: The simulated recharge components (Figure 2b) are modeled as “unidirectional” from the “top” to the “bottom” and into the pipe network. In this model, it is not possible to account for a change in this direction, representing, for example, a pressurized conduit recharging the fissured matrix domain, as has been applied in a fully distributed modeling environment (Reimann et al., 2011) or lumped modeling environment (Mazzilli et al., 2017). The inclusion of such dynamics may improve the overall modeling results in the high‐frequency range.

Finally, this presented approach was developed for studying the intrinsic heterogeneities of a karst aquifer based on spring hydrographs. The results of the simulated and observed time series improve the understanding of internal dynamics of the studied catchment, and they are applicable to other karst systems. If available, the approach could be coupled with the analysis of well hydrographs to understand spatial patterns in the system. With regard to a wider context, the approach is likely to be applicable for studying the signature of climatic influences on environmental systems by means of time series analysis and numerical modeling, including, for example, the field of surface water hydrology and quantification of different contributions to runoff, or assessing the induced impacts of land use changes on measurable environmental systems. In addition, the here presented approach may support assessing the performance of different numerical models, such as reservoir models, neural networks, or distributed groundwater flow models.

## Conflict of Interest

There are no real or perceived financial conflicts of interests of any of the authors.

## Data Availability

The shapefile of the geologic map 1: 100,000 can be found on the GSI website (https://www.gsi.ie/en‐ie/data‐and‐maps/Pages/Bedrock.aspx). The data necessary to reproduce the work are available in the public repository of Zenodo (http://doi.org/10.5072/zenodo.651336), including observed rainfall and spring discharge, observed temperature and estimated potential evapotranspiration, and all simulated time series. Further, it provides the R code for computing the power spectrum, produced by Nicolas Massei (University of Rouen, France) and modified by Lea Duran.
